# Subtyping of high grade serous ovarian carcinoma: histopathological and immunohistochemical approach

**DOI:** 10.1186/s43046-022-00104-9

**Published:** 2022-02-09

**Authors:** Marwa Khashaba, Mohamed Fawzy, Azza Abdel-Aziz, Ghada Eladawei, Reham Nagib

**Affiliations:** 1 Port Said University, Portsaid, Egypt; 2 Mansoura University, Mansoura, Egypt

**Keywords:** Papillary serous carcinoma, Prognosis, Mesenchymal, Proliferative, Immunoreactive

## Abstract

**Background:**

High-grade serous ovarian carcinoma (HGSOC) is classified into four molecular subtypes; mesenchymal, proliferative, immunoreactive, and differentiated, with suggested different prognosis. Addressing the presence of histopathological and immunohistochemical differences in HGSOC that parallel the molecular subtypes can help in tailoring the management protocol to improve therapeutic response and patient outcome.

**Methods:**

This retrospective study was conducted on 85 specimens for cases of HGSOC. Cases were classified according to histopathological findings into mesenchymal, proliferative, immunoreactive, and differentiated subtypes. Cases were immunostained with ER, PR, Ki67, CD8, E-cadherin, and vimentin.

**Results:**

By applying histopathological data, cases were subdivided into 4 groups; mesenchymal type represented by 25 cases, proliferative type which included 14 cases, the immunoreactive type included 14 cases, and differentiated type represented by 32 cases; 13 of them had SET features and 19 had papillary architectural features. A significant correlation was found between Ki67 and proliferative subtype, as well as between CD8 and immunoreactive subtype. ER showed significantly higher expression in proliferative subtype in the group treated by primary debulking. CD8 showed a significant correlation with solid endometroid transitional (SET) pattern in the group that underwent interval debulking. In terms of prognosis, the shortest median progression-free survival (PFS) was for mesenchymal subtype, while the longest median PFS was for differentiated subtype with SET architectural pattern with statistically significant correlation. No correlation was found between any of the studied parameters and overall survival.

**Conclusion:**

Histopathological features and immunohistochemistry can help to stratify HGSOC into prognostic distinct groups.

## Background

High grade serous ovarian carcinoma (HGSOC) is one of the most lethal gynecological malignancies worldwide with increasing incidence [1]. Interestingly, HGSOC does not behave in the same way regarding chemoresponsivness, tumor recurrence, and overall survival rates highlighting the heterogeneous nature and possibility for the presence of different molecular subtypes [2]. This was confirmed by The Cancer Genome Atlas (TCGA) project that revealed four molecular subtypes; mesenchymal, proliferative, immunoreactive, and differentiated, with suggested different prognosis. After this classification has been established, few studies were available in the literature addressing the relation between this classification and patient prognosis. Studies reported that the mesenchymal and proliferative subtypes had the worst overall survival, while the immunoreactive pattern showed a better prognosis, and the differentiated type had an intermediate prognosis [3, 4].

According to TCGA study, the mesenchymal type is characterized by gene signatures of increased stromal components, epithelial mesenchymal transition (EMT), and angiogenesis. On the other hand, a subtype with a good prognosis, immunoreactive, is characterized by upregulation of genes of immune cell activation. As its name reflects, the differentiated subtype displays gene signatures of a more mature stage of development with high expression of ovarian tumor markers (MUC1 and MUC16) along with the expression of the secretory fallopian tube maker SLPI. Also observed in this subtype is the low expression of stromal reaction genes. On the contrary is the proliferative subtype which is characterized by increased expression of proliferative markers as PCNA and increased WNT/β-catenin signaling with decreased E-cadherin expression. This subtype is associated with a low serum level of CEA in relation to other types [5, 6].

However, molecular genetic testing is neither rapid nor cheap to be adopted in daily practice. Therefore, addressing the presence of histopathological and immunohistochemical differences in HGSOC that parallel the molecular subtypes can help in tailoring the management protocol hoping to improve therapeutic response and patient outcome [7]. To the best of our knowledge, few studies in the literature were concerned with this issue with conflicting results [7, 8]. Consequently, this study was carried out trying to answer the question of whether histopathological data & immunohistochemistry (IHC) can help in subtyping of HGSOC into prognostically distinct groups.

## Methods

This retrospective study was conducted on 85 specimens for cases of HGOSC and retrieved from archives of the surgical pathology lab at Oncology Center & University Hospitals. The clinical data of those 85 patients who were treated at the Clinical Oncology and Nuclear Medicine Department were revised. The study was approved by the institutional ethical committeeMD/17.01.96

**For histopathological evaluation** whole tissue hematoxylin & eosin (H&E) stained sections were reviewed to assess histopathological parameters, including architectural pattern, mitotic count, stromal cellularity, tumor infiltrating lymphocytes (TILs), and lymphovascular emboli. None of the cases showed fallopian tube involvement and no STIC lesion was seen in any of the examined cases. Cases were categorized into 4 subtypes parallel to the molecularly classified groups. These subtypes are mesenchymal, proliferative, immunoreactive, and differentiated either with a solid endometroid transitiona (SET) pattern or with a papillary pattern.

Tumors with a cellular stromal reaction in >10% of tumor tissue were categorized as tumors with mesenchymal patterns [7].

Tumors were categorized as the proliferative type when the mitotic count was >30/10 HPF [9]. Tumors were defined as immunoreactive type when lymphocytes infiltrating tumor nests >20 /HPF [10].

**Fig. 1:**
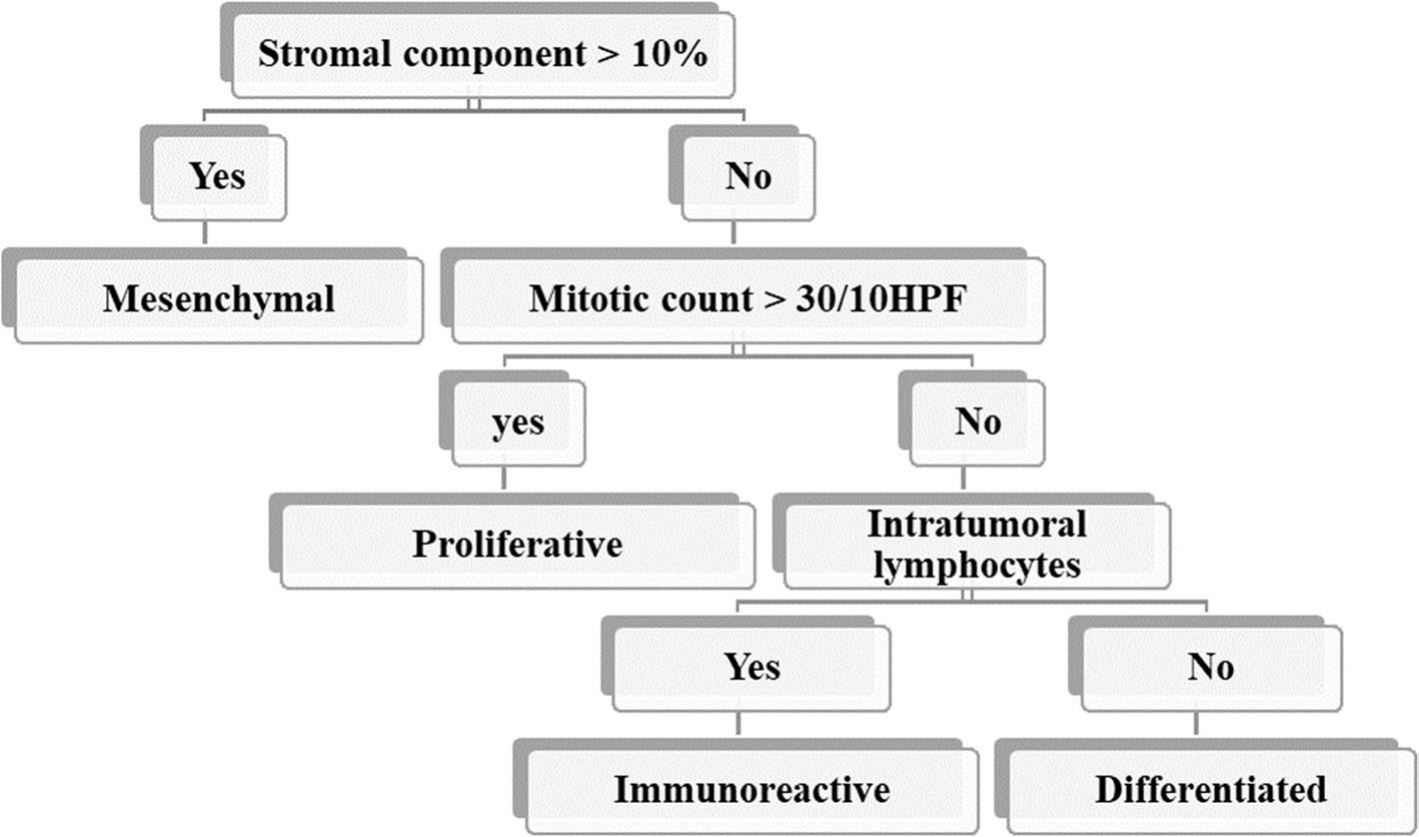
An algorithm for histopathological classification of cases of HGSC

Cases displayed diffuse, insular, trabecular, cribriform, and punched out microspaces or pseudoendometrioid glandular morphology in more than 25% of the examined sections were considered in the SET category. Otherwise, tumors were included in the papillary and micropapillary categories [11]. Cases were classified according to an algorithm (Fig. 1), where the stromal component was first to evaluate, the mitotic count was the next parameter, and the third parameter was the intratumoral lymphocytic count. Lastly, after exclusion of all criteria, cases were classified as differentiated followed by subclassification according to the pattern [7]

**For immunohistochemistry**, tissue microarray (TMA) blocks were constructed using the pencil tip method adopted from Shebl et al. (2011) where each case was represented by three cores in the recipient block. The 4-um-thick sections were cut on coated glass slides, deparaffinized, then rehydrated with xylene and graded alcohols [12]. Antigen retrieval was performed at 98°C in a 10-mM sodium citrate buffer PH 9 for 40 min. Sections were counterstained with Mayer’s hematoxylin. Slides were immunostained with markers (ki67, CD8, ER, PR, vimentin and E-cadherin). All were mouse monoclonal antibodies, ready to use, (DakoCytomation, Glostrup, Denmark). All immune stains were performed on the primary ovarian tumor not on metastases. Positive controls were prepared for each marker as Gastric tissue for ki67, tonsillar tissue for CD8, leiomyoma for ER & PR, endothelial cells for vimentin as internal controls, and normal breast tissue for E-cadherin).

### Interpretation of IHC results

Ki-67 is considered high when it is expressed in > 40% of tumor cells nuclei, and < 40 % was considered low expression [13]. The expression of Ki67 was scored by selecting the hotspot areas. Also, each core was screened for a hotspot of CD8+ TILs at (x20) power, within each hotspot; 3 high power fields at 400-× magnification were evaluated and average number of TILs was taken. Only CD8+ TILs within the epithelial component of the tumor (tumor islets) were considered avoiding interpretation of any CD8+cells within the stroma, areas of necrosis, or inflammation. A score was defined based on CD8+ TIL counts per high-powered field: low (<20 TILs/HPF) and high (≥20 TILs/HPF) [10].

ER and PR levels evaluated as positive nuclear staining of any intensity in > 10% of tumor cells as cut off point [14].

Vimentin expression was evaluated subjectively where expression in > 30% of tumor cells is considered high and in <of 30% of tumor cells comprises low expression [15].

Assessment of E-cadherin expression used 10% of tumor cells as cutoff point. E-cadherin was considered positive when ≥ 10% of tumor cells exhibited strong membranous immunoreactivity. However, it was considered negative when exhibited strong membranous and/ cytoplasmic staining but in <10% of tumor cells or exhibited faint and incomplete expression [16, 17]. All histopathological and IHC data were recorded by 2 independent pathologists blinded to the prognostic data.

### Statistical analysis and data interpretation

Data were fed to the computer and analyzed using IBM SPSS software package version 22.0. Qualitative data were described using number and percent. Quantitative data were described using mean, standard deviation for parametric data after testing normality using Shapiro–Wilk test. Significance of the obtained results was judged at the (0.05) level. Chi-square test for comparison of 2 or more groups with correction for chi-square was used if 25% of cells or more have count less than 5 (by Monte Carlo and Fischer Exact tests).Kaplan-Meier test was used to calculate overall survival and disease-free survival with using log rank χ^2^ to detect effect of risk factors affecting survival.

## Results

### Clinical data

The mean age for studied cases was 52.78±8.63 years. According to the management protocol, 46 cases underwent primary debulking while 39 underwent interval debulking with a statistically significant difference between the two protocols regarding the patient response. All clinical data were illustrated in (Table 1). Patient response was carefully analyzed as highlighted in Table 2.

**Table 1:** Clinical data of studied cases (No. 85)

Clinical parameter	Total number =85	
**Age/years, mean±SD (min-max)**	52.78±8.63 (30.00–71.0)	
**Ascites** ***N*=57**		
**Negative**	25	43.9
**Positive**	32	56.1
**CA 125 median (min-max)**	441.0 (52.0–13300.0)	
**Stage**	*N*=85	%
**I**	12	14.1
**II**	5	5.9
**III**	67	78.8
**IV**	1	1.2
**Treatment type**		
**Primary DS followed by adjuvant therapy**	46	54.1
**NACT & IDS then adjuvant therapy**	39	45.9
**Neo-adjuvant therapy type**		
**Taxol carbo**	37	94.9
**Others**	2	5.1
**Adjuvant therapy type**		
**Taxol carbo**	76	89.4
**Others**	9	10.6
**Total cycles**		
**Median (min-max)**	6.0 (1.0–15.0)	
**Residual after debulking surgery**	16	18.8%

**Table 2:** Treatment response among studied cases

Treatment response	***N***= 46(%) Primary debulking	***N***=39(%) Interval debulking	Test of significance
• **Regression**	14 (30.4)	7 (17.9)	χ^2^=8.12 *P*=0.04*
• **Recurrence**	16 (34.8)	12 (30.8)	
• **Metastasis**	11 (23.9)	6 (15.4)	
• **Progression**	5 (10.9)	14 (35.9)	

#### Histopathological data

**Fig. 2:**
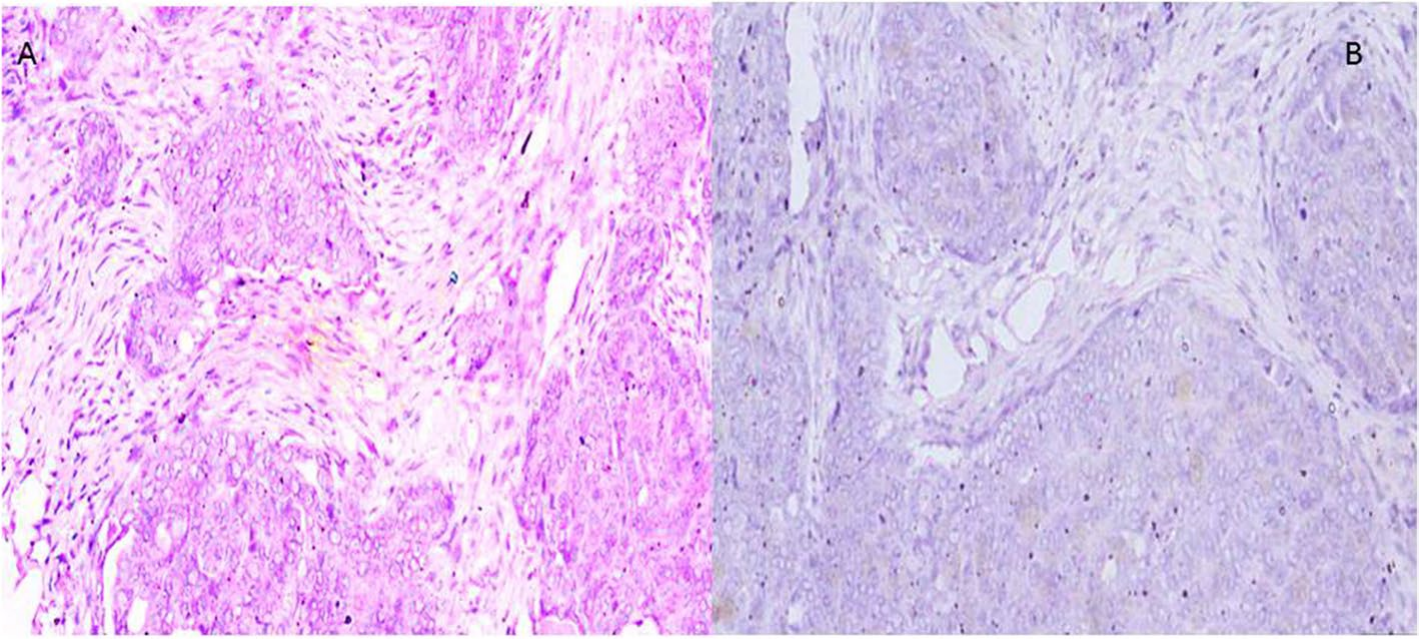
**x400 A** H&E photomicrograph of mesenchymal subtype of HGSC where cellular stroma around tumor sheets is seen. **B** Negative E-cadherin expression in same tumor type

**Fig. 3:**
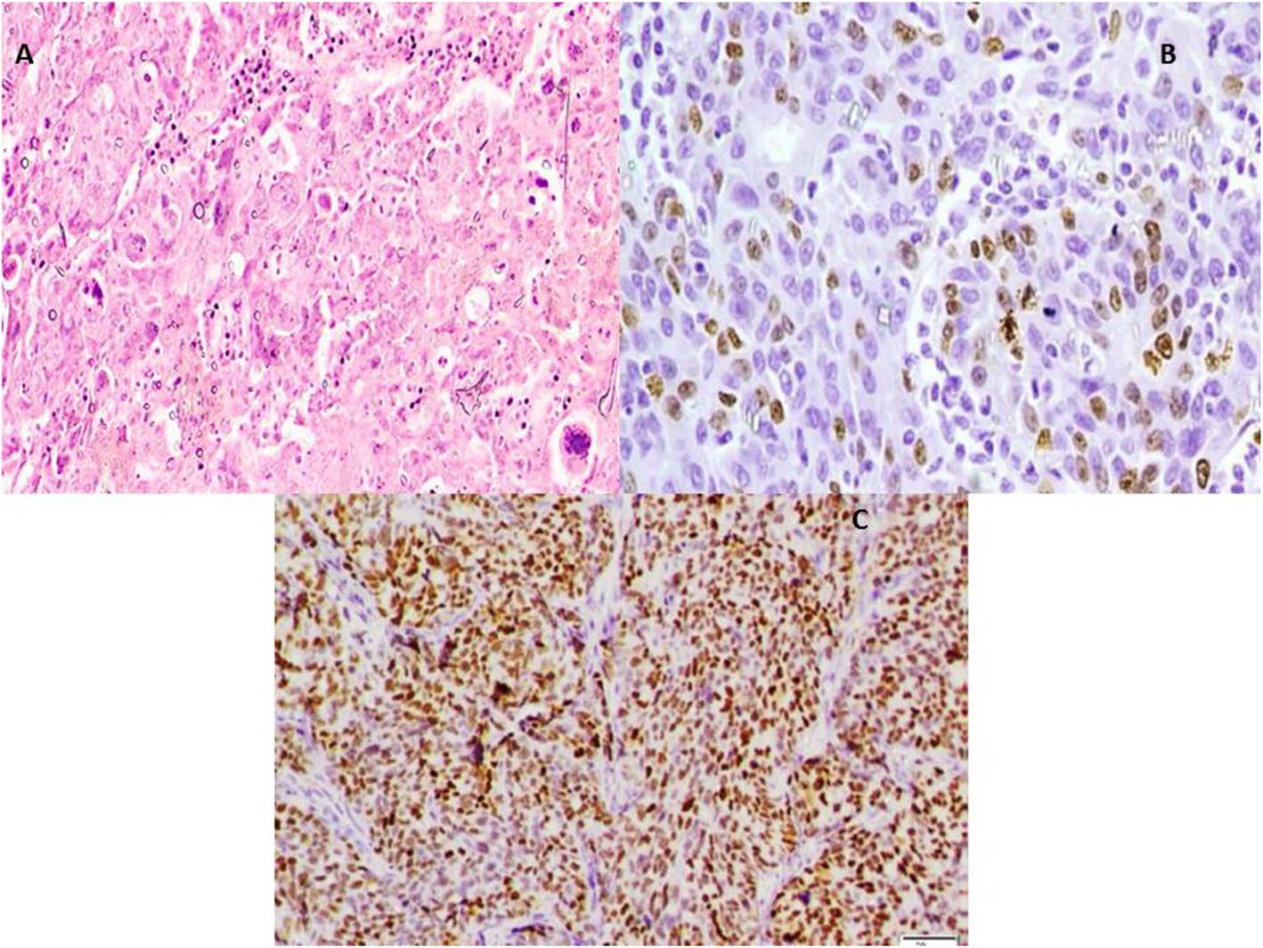
**x400 A** H&E photomicrograph of proliferative subtype of HGSC showing frequent mitotic figures. **B** Nuclear expression of Ki67 in > 25% of tumor cell nuclei. **C** Diffuse strong nuclear expression of ER in the same case

**Fig. 4:**
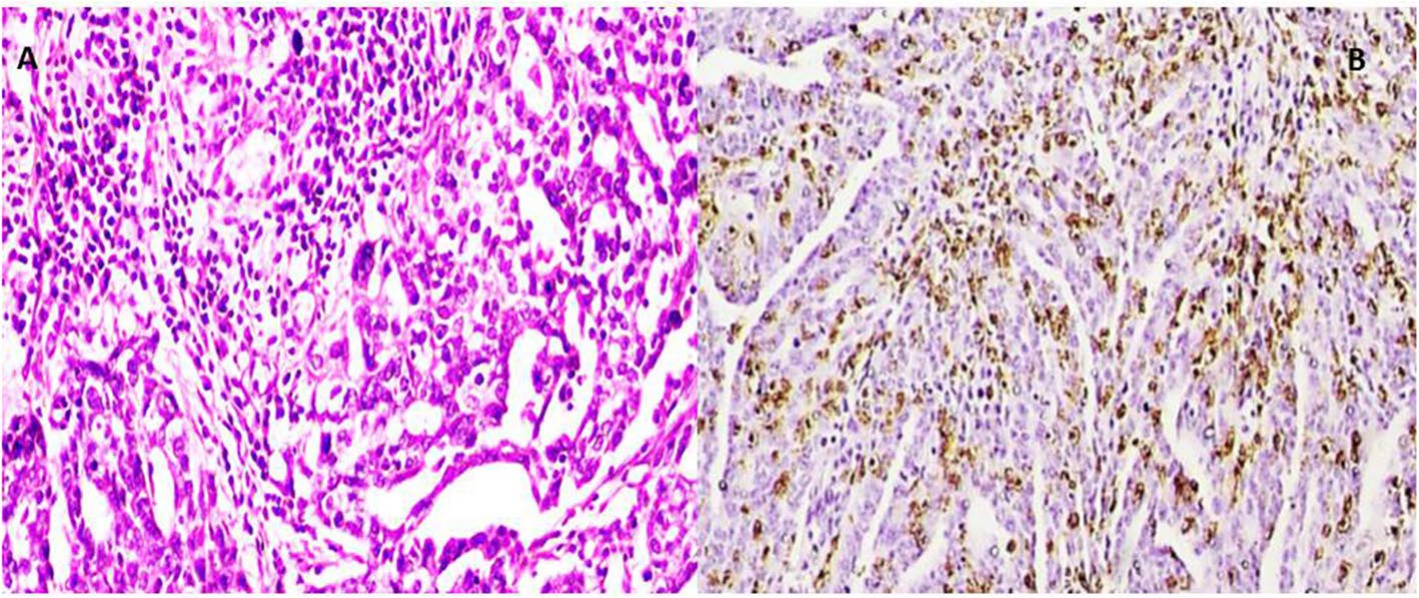
x400 A H&E photomicrograph of immunoreactive subtype of HGSC, lymphocyte-infiltrating tumor nests >20 /HPF. **B** These lymphocytes are highlighted by CD8

**Fig. 5:**
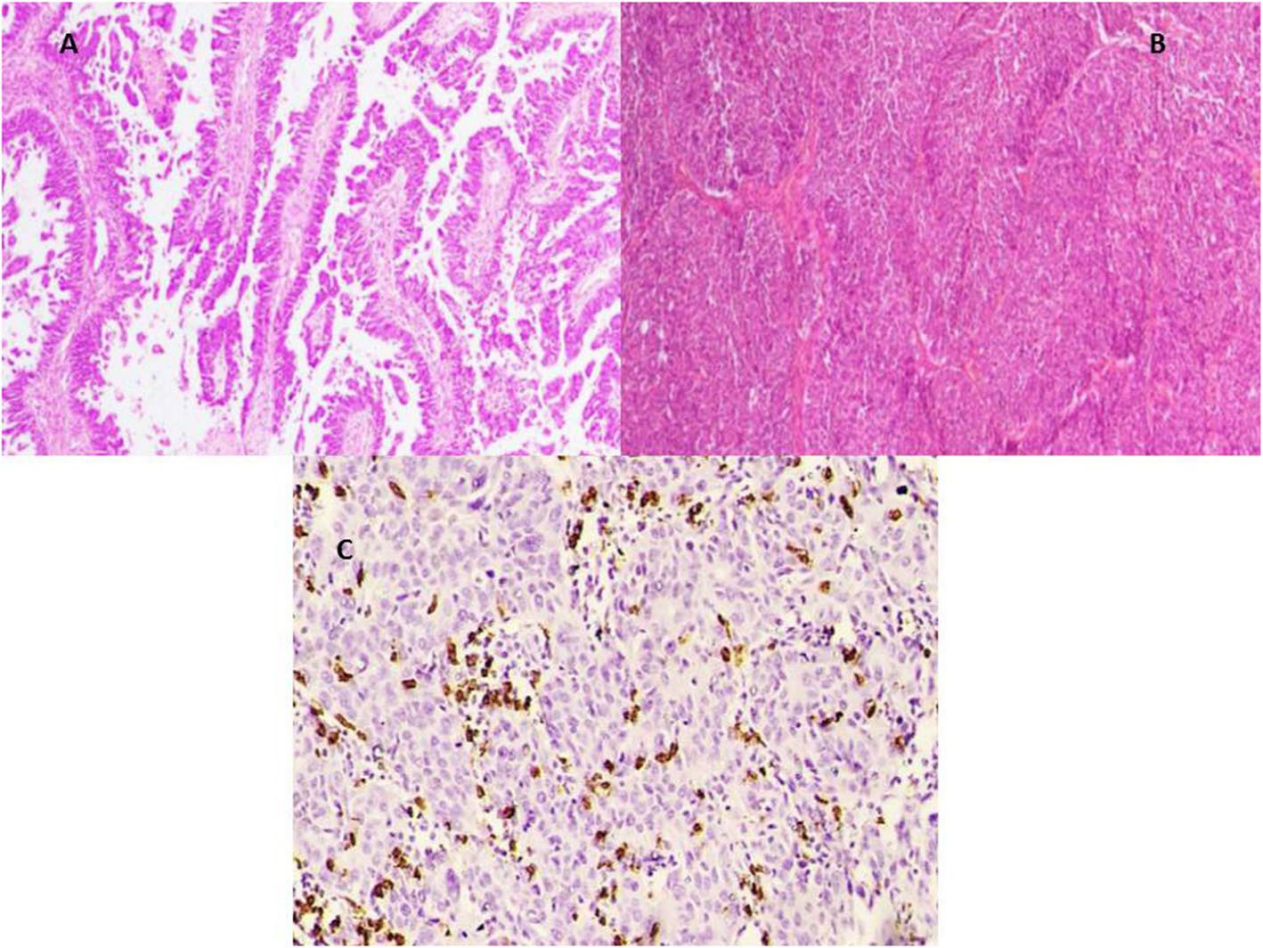
**A** H&E. Photomicrograph of differentiated subtype of HGSC formed of papillary structures (x200). **B** H&E. Photomicrograph of differentiated subtype of HGSC with SET pattern (x200). **C** CD8 highlighting intratumoral lymphocytes between the sheets of HGSC with a SET pattern

All cases revealed high-grade cytological features including pleomorphism with prominent nucleoli and frequent mitotic figures > 12 /HPF. This was coupled with variable architectural patterns as complex papillary structures, irregular glands with slit-like lumina or pseudoendometrioid morphology, as well as solid sheets. In addition to morphology, WT1 &/or P53 mutant phenotype confirmed the diagnosis of HGSOC. Cases were subdivided into 4 groups including mesenchymal type (Fig. 2) represented by 25 cases, proliferative type (Fig. 3) which included other 14 cases, immunoreactive type (Fig. 4) that included 14 cases and differentiated type (Fig. 5) represented by 32 cases; 13 of them had SET features and 19 had papillary architectural features (Table 3).

**Table 3:** Histopathological types of the studied cases

Histopathological type		***N***=85	%
**Mesenchymal type**		**25**	**29.4**
**Proliferative type**		**14**	**16.5**
**Immunoreactive type**		**14**	**16.5**
**Differentiated type**	**With SET features**	**13**	**15.3**
	**With Papillary features**	**19**	**22.4**

#### Immunohistochemical data

When correlated with the histopathological subtype, the highest expression of Ki 67 was observed in proliferative type (100% of cases), and the lowest expression was observed in all cases with differentiated subtype either with SET features or papillary features. CD8 was highly expressed in 100% of cases with immune reactive phenotype, while it had the lowest expression in all cases with proliferative subtype. The positive expression of ER was observed in most cases of proliferative subtype (64%); however, its negative expression was observed mainly in cases with differentiated morphology that exhibit papillary features (78%). PR and E-cadherin were both negative in most of the studied cases including all subtypes. Regarding vimentin IHC, the lowest expression of vimentin was observed in proliferative cases (64%), while it was of highest expression in mesenchymal type (56%) (Table 4).

**Table 4:** Expression of immunohistochemical markers in relation to histopathological types

	Mesenchymal type ***N***=25(%)	Proliferative type ***N***=14(%)	Immunoreactive type ***N***=14(%)	Differentiated type ***N***=32(%)		Test of significance
				SET features ***N***=13(%)	Papillary features ***N***=19(%)	
**Ki 67**						
Low	13 (52.0)	0 (0.0)	8 (57.1)	13 (100.0)	19 (100.0)	**MC**
High	12 (48.0)	14 (100.0)	6 (42.9)	0 (0.0)	0 (0.0)	*P*<0.001*
**CD8**						
Low	15 (60.0)	14 (100.0)	0 (0.0)	13 (100.0)	19 (100.0)	**MC**
High	10 (40.0)	0 (0.0)	14 (100.0)	0 (0.0)	0 (0.0)	*P*<0.001*
**ER**						
Negative	11 (44.0)	5 (35.7)	7 (50.0)	6 (46.2)	15 (78.9)	χ^2^=7.85
Positive	14 (56.0)	9 (64.3)	7 (50.0)	7 (53.8)	4 (21.1)	*P*=0.097
**PR**						
Negative	22 (88.0)	11 (78.6)	14 (100.0)	9 (69.2)	18 (94.7)	MC
Positive	3 (12.0)	3 (21.4)	0 (0.0)	4 (30.8)	1 (5.3)	*P*=0.105
**Vimentin**						
Low	11 (44.0)	9 (64.3)	8 (57.1)	6 (46.2)	12 (63.2)	χ^2^=2.63
High	14 (56.0)	5 (35.7)	6 (42.9)	7 (53.8)	7 (36.8)	*P*=0.623
**E-cadherin**						
Negative	24 (96.0)	12 (85.7)	13 (92.9)	11 (84.6)	18 (94.7)	MC
Positive	1 (4.0)	2 (14.3)	1 (7.1)	2 (15.4)	1 (5.3)	*P*=0.66

#### Correlation between immunohistochemical markers and clinico-pathological data

Cases were categorized into 2 groups according to their treatment protocol; those who underwent primary debulking (46 cases) and those who underwent interval debulking (39 cases).

### In the group of primary debulking

A statistically significant association was found between high Ki67 and proliferative subtype (*P*<0.001). Additionally, there was a statistically significant association between immunoreactive subtype and high CD8 expression (*P*<0.001). In addition, positive ER was significantly associated with proliferative subtype (*P*=0.04). On the other hand, disease progression and relapse were significantly associated with negative PR (*P*=0.01) and negative E-cadherin (*P*=0.01). Negative PR expression was also associated with advanced-stage disease (*P*=0.03). Vimentin expression did not show an association with any histopathological parameter (Table 5).

**Table 5:** Immunohistochemical data and clinicopathological parameters for cases underwent primary debulking surgery

**Group 1** ***N*=46**	**Ki 67**		**CD8**		**ER**	
	**Low**	**High**	**Low**	**High**	**Negative**	**Positive**
**Lymphocytes**						
**<20/HPF**	17 (85)	17 (65.4)	34 (100)	0 (0.0)	16 (80)	18 (69.2)
**>20/HPF**	3 (15)	9 (34.6)	0 (0.0)	12 (100.0)	4 (20)	8 (30.8)
**Test of significance**	χ^2^=2.26 *P*=0.13		FET *P*<0.001*		χ^2^=0.68 *P*=0.41	
**Stroma**						
**<10%**	15 (75)	18 (69.2)	25 (73.5)	8 (66.7)	16 (80)	17 (65.4)
**>10%**	5 (25)	8 (30.8)	9 (26.5)	4 (33.3)	4 (20)	9 (34.6)
**Test of significance**	χ^2^=0.186 *P*=0.67		χ^2^=0.21 *P*=0.65		χ^2^=1.19 *P*=0.27	
**Mitosis**						
**<30/10HPF**	20 (100)	0	17 (50)	3 (25)	12 (60)	8 (30)
**>30/10HPF**	0	26 (100)	17 (50)	9 (75)	8 (40)	18 (69.2)
**Test of significance**	FET *P*<0.001*		χ^2^=2.26 *P*=0.13		χ^2^=3.93 *P*=0.04*	
**Architecture**						
**SET features**	11 (55)	17 (65.4)	19 (55.9)	9 (75)	10 (50)	18 (69.2)
**Papillary features**	9 (45)	9 (34.6)	15 (44.1)	3 (25)	10 (50)	8 (30.8)
**Test of significance**	χ^2^=0.51 *P*=0.47		χ^2^=1.36 *P*=0.24		χ^2^=1.76 *P*=0.18	
**Treatment response**						
**Regression**	4 (20.0)	8 (30.8)	9 (26.5)	3 (25.0)	5 (25.0)	7 (26.9)
**Progression**	16 (80.0)	18 (69.2)	25 (73.5)	9 (75.0)	15 (75.0)	19 (73.1)
**Test of significance**	χ^2^=0.680 *P*=0.51		χ^2^=0.01 *P*=0.92		χ^2^=0.02 *P*=0.88	
**Stage**						
**I & II**	5 (25.0)	4 (15.4)	6 (17.6)	3 (25.0)	3 (15.0)	6 (23.1)
**III & IV**	15 (75.0)	22 (84.6)	28 (82.4)	9 (75.0)	17 (85.0)	20 (76.9)
**Test of significance**	χ^2^=0.66 *P*=0.42		χ^2^=0.305 *P*=0.58		χ^2^=0.469 *P*=0.49	
**Group 1** ***N*=46**	**PR**		**Vimentin**		**E Cadherin**	
	**Negative**	**Positive**	**Low**	**High**	**Negative**	**Positive**
**Lymphocytes**						
**<20/HPF**	26 (68.4)	8 (100)	20 (76.9)	14 (70)	32 (74.4)	2 (66.7)
**>20/HPF**	12 (31.6)	0 (0)	6 (23.1)	6 (30)	11 (25.6)	1 (33.3)
**Test of significance**	χ^2^=3.42 *P*=0.06		χ^2^=0.28 *P*=0.59		FET *P*=1.0	
**Stroma**						
**<10%**	27 (71.1)	6 (75)	21 (80.8)	12 (60)	31 (72.1)	2 (66.7)
**>10%**	11 (28.9)	2 (25)	5 (19.2)	8 (40)	12 (27.9)	1 (33.3)
**Test of significance**	χ^2^=0.051 *P*=0.82		χ^2^=2.41 *P*=0.12		FET *P*=1.0	
**Mitosis**						
**<30/10HPF**	16 (42.1)	4 (50)	11 (42.3)	9 (45)	20 (46.5)	0
**>30/10HPF**	22 (57.9)	4 (50)	15 (57.7)	11 (55)	23 (53.5)	3 (100)
**Test of significance**	FET *P*=0.71		χ^2^=0.03 *P*=0.86		χ^2^=2.46 *P*=0.12	
**Architecture**						
**SET features**	21 (55.3)	7 (87.5)	17 (65.4)	11 (55)	26 (60.5)	2 (66.7)
**Papillary features**	17 (44.7)	1 (12.5)	9 (34.6)	9 (45)	17 (39.5)	1 (33.3)
**Test of significance**	χ^2^=2.88 *P*=0.09		χ^2^=0.51 *P*=0.47		FET *P*=1.0	
**Treatment response**						
**Regression**	7 (18.4)	5 (62.5)	5 (19.2)	7 (35)	9 (20.9)	3 (100.0)
**Progression**	31 (81.6)	3 (37.5)	21 (80.8)	13 (65)	34 (79.1)	0 (0.0)
**Test of significance**	χ^2^=6.66 *P*=0.01*		χ^2^=1.46 *P*=0.22		FET *P*=0.01*	
**Stage**						
**I & II**	5 (13.2)	4 (50.0)	5 (19.2)	4 (20.0)	7 (16.3)	2 (66.7)
**III & IV**	33 (86.8)	4 (50.0)	21 (80.8)	16 (80.0)	36 (83.7)	1 (33.3)
**Test of significance**	FET *P*=0.03*		χ^2^=0.004 *P*=0.948		FET *P*=0.09	

*FET* Fisher’s exact test, *MC* Monte Carlo

### In the interval debulking group

A statistically significant association was found between high Ki67 and proliferative subtype (*P*<0.001). In addition, there was a statistically significant association between immunoreactive subtype and CD8 expression (*P*<0.001). Differentiated subtype with SET features showed a statistically significant association with high CD8 expression (*P*=0.04) (Table 6).

**Table 6:** Immunohistochemical data and clinicopathological parameters for cases underwent interval debulking surgery

**Group 2** ***N*=39**	**Ki 67**		**CD8**		**ER**	
	**Low**	**High**	**Low**	**High**	**Negative**	**Positive**
**Lymphocytes**						
**<20/HPF**	24 (72.7)	3 (50.0)	27 (100.0)	0	16 (66.7)	11 (73.3)
**>20/HPF**	9 (27.3)	3 (50.0)	0 (0.0)	12 (100)	8 (33.3)	4 (26.7)
**Test of significance**	FET *P*=0.35		FET *P*<0.001*		χ^2^=0.19 *P*=0.66	
**Stroma**						
**<10%**	25 (75.8)	2 (33.3)	21 (77.8)	6 (50)	17 (70.8)	10 (66.7)
**>10%**	8 (24.2)	4 (66.7)	6 (22.2)	6 (50)	7 (29.2)	5 (33.3)
**Test of significance**	FET *P*=0.06		χ^2^=3.01 *P*=0.08		χ^2^=0.08 *P*=0.78	
**Mitosis**						
**<30/10HPF**	33 (100.0)	0 (0.0)	24 (88.9)	9 (75)	22 (91.7)	11 (73.3)
**>30/10HPF**	0 (0.0)	6 (100)	3 (11.1)	3 (25)	2 (8.3)	4 (26.7)
**Test of significance**	FET *P*<0.001*		FET *P*=0.34		FET *P*=0.18	
**Architecture**						
**SET features**	15 (45.5)	5 (83.3)	11 (40.7)	9 (75)	10 (41.7)	10 (66.7)
**Papillary features**	18 (54.5)	1 (16.7)	16 (59.3)	3 (25)	14 (58.3)	5 (33.3)
**Test of significance**	χ^2^=2.92 *P*=0.09		χ^2^=3.9 *P*=0.04*		χ^2^=2.31 *P*=0.13	
**Treatment response**						
**Regression**	7 (21.2)	0 (0.0)	5 (18.5)	2 (16.7)	4 (16.7)	3 (20.0)
**Progression**	26 (78.8)	6 (100.0)	22 (81.5)	10 (83.3)	20 (83.3)	12 (80.0)
**Test of significance**	χ^2^=1.55 *P*=0.21		χ^2^=0.02 *P*=0.89		FET *P*=1.0	
**Stage**						
**I & II**	7 (21.2)	1 (16.7)	5 (18.5)	3 (25)	4 (16.7)	4 (26.7)
**III & IV**	26 (78.8)	5 (83.3)	22 (81.5)	9 (75)	20 (83.3)	11 (73.3)
**Test of significance**	χ^2^=0.06 *P*=0.80		χ^2^=0.214 *P*=0.64		FET *P*=0.68	
**Group 2** ***N*=39**	**PR**		**Vimentin**		**E-cadherin**	
	**Negative**	**Positive**	**Low**	**High**	**Negative**	**Positive**
**Lymphocytes**						
**<20/HPF**	24 (66.7)	3 (100)	15 (75)	12 (63.2)	24 (68.6)	3 (75)
**>20/HPF**	12 (33.3)	0	5 (25)	7 (36.8)	11 (31.4)	1 (25)
**Test of significance**	FET *P*=0.23		χ^2^=0.64 *P*=0.42		FET *P*=1.0	
**Stroma**						
**<10%**	25 (69.4)	2 (66.7)	14 (70)	13 (68.4)	23 (65.7)	4 (100)
**>10%**	11 (30.6)	1 (33.3)	6 (30)	6 (31.6)	12 (34.3)	0 (0.0)
**Test of significance**	FET *P*=1.0		χ^2^=0.01 *P*=0.92		FET *P*=0.29	
**Mitosis**						
**<30/10HPF**	30 (83.3)	3 (100)	19 (95)	14 (73.7)	29 (82.9)	4 (100)
**>30/10HPF**	6 (16.7)	0	1 (5)	5 (26.3)	6 (17.1)	0 (0.0
**Test of significance**	FET *P*=1.0		χ^2^=3.4 *P*=0.07		FET *P*=1.0	
**Architecture**						
**SET features**	19 (52.8)	1 (33.3)	9 (45)	11 (57.9)	17 (48.6)	3 (75)
**Papillary features**	17 (47.2)	2 (66.7)	11 (55)	8 (42.1)	18 (51.4)	1 (25)
**Test of significance**	FET *P*=0.61		χ^2^=0.65 *P*=0.42		FET *P*=0.61	
**Treatment response**						
**Regression**	6 (16.7)	1 (33.3)	5 (25)	2 (10.5)	5 (14.3)	2 (50.0)
**Progression**	30 (83.3)	2 (66.7)	15 (75)	17 (89.5)	30 (85.7)	2 (50.0)
**Test of significance**	FET *P*=0.45		χ^2^=1.38 *P*=0.239		FET *P*=0.14	
**Stage**						
**I & II**	7 (19.4)	1 (33.3)	5 (25)	3 (15.8)	7 (20.0)	1 (25)
**III & IV**	29 (80.6)	2 (66.7)	15 (75)	16 (84.2)	28 (80.0)	2 (66.7)
**Test of significance**	FET *P*=0.508		χ^2^=0.507 *P*=0.47		FET *P*=1.0	

*FET* Fischer’s exact test, *MC* Monte Carlo

### Correlation of studied histopathological and immunohistochemical parameters with progression-free survival

**Fig. 6:**
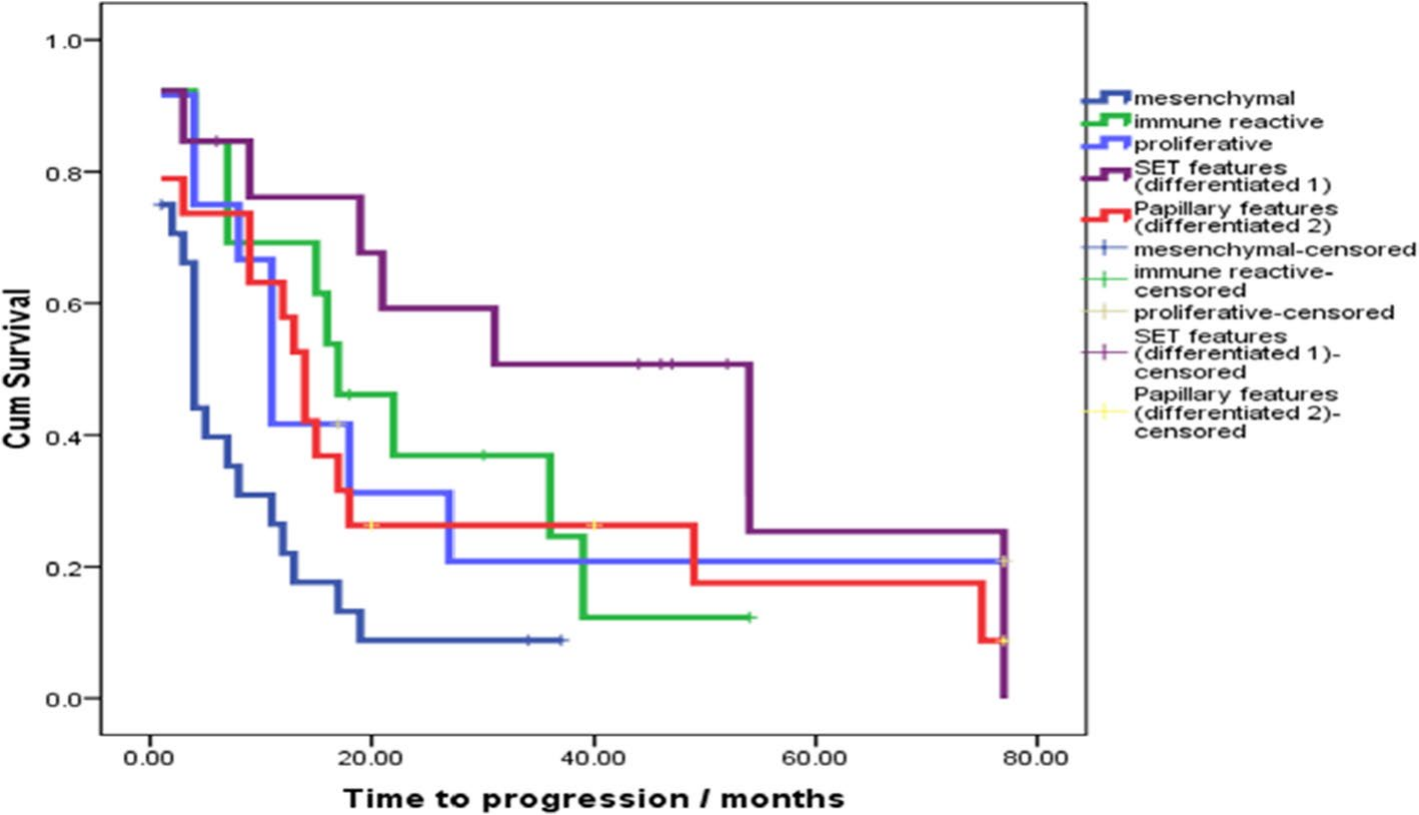
The histopathological type is a significant factor for PFS. *P*=0.008

A univariate analysis was done to test the impact of histopathological types and studied immunohistochemical markers on progression free survival (PFS). The histopathological type could be detected as a significant factor (*P*=0.008) for PFS, where the shortest median PFS was for mesenchymal subtype, while the longest median PFS was for differentiated subtype with the SET architectural pattern (Fig. 6).

**Fig. 7:**
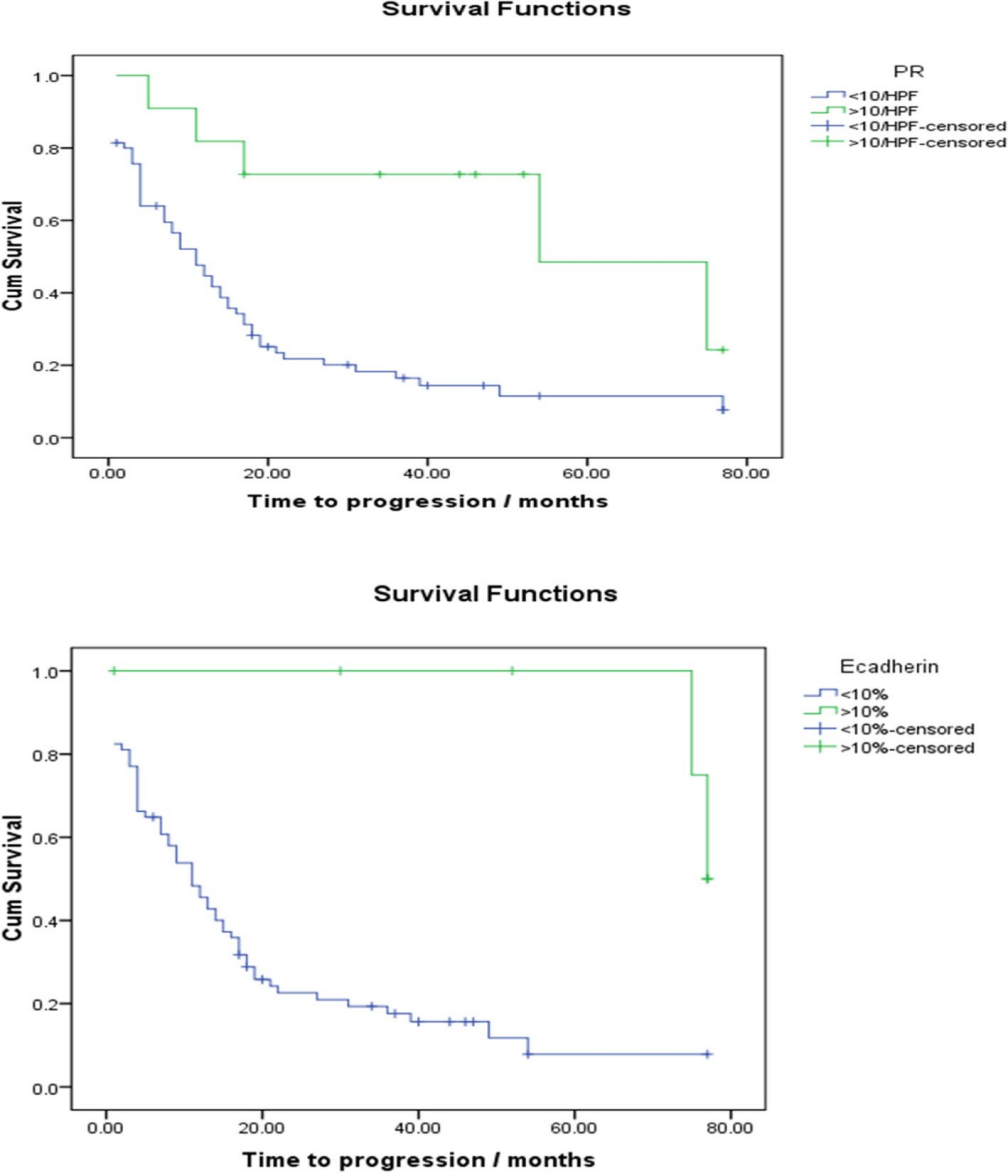
Immunohistochemical markers significantly associated with PFS

Additionally, longer PFS was significantly correlated with positive PR expression (*P*=0.003), positive E-cadherin *P*=0.001, absence of lympho-vascular invasion (LVI) (*P*=0.002), cases underwent 1ry debulking protocol (*P*=0.004), as well as Taxol carbo chemotherapeutic agents (*P*=0.001) (Fig. 7).

### Correlation of studied histopathological and immunohistochemical parameters with overall survival

In another univariate analysis with overall survival (OS), none of the studied four histopathological types, immunohistochemical markers, LVI, nor stage showed a statistically significant impact on OS. Only the treatment protocol showed a significant difference where cases that underwent primary debulking surgery followed by adjuvant therapy had a longer median OS (56.8 months) than cases that received neoadjuvant therapy (NACT) followed by interval debulking surgery (IDS) (44.27 months) with a statistically significant difference between them (*P*=0.006). However, it was observed that cases with low vimentin expression had a longer median overall survival (92 months) compared to cases with high vimentin expression (34 months) with no statistically significant difference. In addition, positive expression of E-cadherin was associated with longer median overall survival (61 months) versus cases with negative expression (47 months) but also with no statistically significant effect. It was observed also that cases at earlier stages had a prolonged OS (51 months), while cases with late stages had the least OS (18 months) but did not reach a statistically significant value. Additionally, The median OS was longer with a statistically significant difference between cases that received taxol carbo (51 months) than cases that received agents other than taxol carbo regimen (20 months) (*P*=0.049).

### Analysis for relapse predictors

Simple model analysis of factors that could predict the disease relapse revealed that advanced stage (*P*=0.037), negative E-cadherin (*P*=0.001), negative PR (*P*=0.006), and the presence of LVI (*P*=0.008) are considered as statistically significant positive predictors (Table 7).

**Table 7:** Analysis for relapse predictors

	Simple model analysis			Multivariate analysis	
	No relapse	Relapse	Test of significance	***P***	AOR (95% CI)
**Stages**			*P*=0.037*	0.409	1.84 (0.432–7.83)
**I & II**	7 (36.8)	10 (15.2)			
**III & IV**	12 (63.2)	56 (84.8)			
**LVI**			*P*=0.008*	0.123	2.73 (0.763–9.753)
**Absent**	14 (73.7)	26 (39.4)			
**Present**	5 (26.3)	40 (60.6)			
**PR**			*P*=0.006*	0.171	3.038 (0.618–14.928)
**Negative**	13 (68.4)	61 (92.4)			
**Positive**	6 (31.6)	5 (7.6)			
**E Cadherin**			*P*=0.001*	0.07	5.69 (0.869–37.32)
**Negative**	14 (73.7)	64 (97.0)			
**Positive**	5 (26.3)	2 (3.0)			

However, multivariate analysis of the same factors revealed that none of them could be detected as an independent prognostic factor for disease relapse either recurrence or metastasis (Table 7).

## Discussion

The more common prevalence of HGSOC among all ovarian epithelial cancers associated with variable patient response to therapy and variable outcome should raise its concern in the scope of research. The Australian Ovarian Cancer Study Group defined four molecular subtypes of HGSOC by gene expression analysis [5] that was validated in the TCGA project [3, 6]. The correlation of these molecular subtypes with histopathological features was addressed in very few studies [7, 8].

Applying the histopathological features described in the literature to the current retrospective study allowed categorization of the cases into 4 groups including mesenchymal type represented by 29% of cases, the proliferative type which included 16.5%, immune reactive type that included other 16.5% of cases, and differentiated type represented by 32% cases; 15% of them had SET features and 22% had papillary architectural features. This study aimed to confirm this subtyping by IHC approach. However, since chemotherapy can alter the IHC expression of markers, cases were divided into 2 groups according to their treatment protocol (primary versus interval debulking) to allow accurate analysis of IHC markers.

In both protocol groups, proliferative subtype showed significant association with Ki67 > 25%. Similarly, the immunoreactive subtype showed a significant association with CD8 (≥20 TILs/HPF). On the contrary, no distinct immunoprofile was found for mesenchymal or differentiated subtypes. These findings were consistent with the study of Popa and colleagues who found that mean Ki-67 expression correlated with the high mitotic count >30/10HPF in their HGSC cases [9]. Additionally, Darb-Esfahani et al. included cases of HGSC that received neoadjuvant chemotherapy and molecularly proved to be immunoreactive subtype. They found >50% of patients with this subtype were characterized by intratumoral enrichment of CD8 positive T cells [18]. In concordance, Murakami et al. reported a significant correlation between CD8 lymphocytes and immunoreactive type [7].

Unexpectedly, ER showed a significant correlation with the proliferative subtype only in cases that underwent primary debulking. To some extent, this is similar to the reports of Popa et al. in their trial when most of their HGSOC cases showed high expression of ki67 and were positive for ER status, but they did not make a correlation between them [9].

Another study by Feng and colleagues applied a hormonal receptor-based classification of HGSC cases and found that cases that were ER-positive had the worst prognosis [14]. In many reports, estrogen is proved to be the driver for ovarian cancer development, promoting proliferation as well as metastases through inhibition of cell to cell adhesion [19–22].

In the same treated patient group, a significant association was found between negativity for PR and E-cadherin in relation to low tumor progression. However, these results need to be validated on a wider scale since this significance may lose its weight due to the negativity of PR and E-cadherin that we found in most of our cases. Despite that, loss or decreased expression of E-cadherin is considered a feature of epithelial-mesenchymal transition (EMT) responsible for tumor dedifferentiation and invasiveness, which plays an important role in tumor progression in epithelial tissues [23].

Considering cases that received neoadjuvant chemotherapy (NACT), a significant correlation was found between high CD8 expression in lymphocytes within tumor cells and the differentiated subtype with a SET pattern. The Association of SET with diffusely dense TILs is known in most cases of BRCA1 mutation [11]. However, the absence of this significant association in our cases that did not receive NACT was concerning. NACT proved their role in the induction of many local changes of the tumor microenvironment including activation of CD4+ and CD8+ T cells. This may be due to the presentation of degraded protein antigens by class I MHC molecules to stimulate CD8+ T lymphocytes to produce proinflammatory cytokines to kill tumor cells [24, 25].

In a trial to find an answer for the study question, whether there are prognostic differences between the histopathological groups, the current work found that most of the cases diagnosed at an advanced stage (III&IV) were mesenchymal and proliferative types but without statistically significant association. This is concordant to Murakami and coworkers who reported a statistically significant association between the mesenchymal type and advanced stage [7]. The limited number of cases in the present study (85) compared to that of Murakami et al. (132) may be the cause for not approaching a statistically significant relation.

Moreover, the present study found a significant correlation between the histopathological subtype and progression-free survival where mesenchymal type had the least PFS and the differentiated type with SET features had the longest PFS. Similar findings were reported by the study of Murakami et al. for the mesenchymal type. On the other hand, they found the immunoreactive type to have the best PFS [7]. In addition, the results of the current study are compatible with the study of Ohsuga et al. who suggested—by using CT—that cases with mesenchymal gene expression subtype had more mesenteric infiltration and wide peritoneal disease and shorter PFS [8].

In a more detailed analysis for factors significantly correlated with longer PFS, high PR expression, positive E-cadherin, absent LVI, and primary debulking were found. These findings are consistent with those reported by Faleiro-Rodrigues et al. (2004), Cho et al. (2006), Modugno et al. (2012), Feng et al. (2016), and Mohanty et al. (2019) [14, 17, 21, 26, 27]. While it could be logical for all these factors to prolong PFS, the effect of high PR expression is explained by the role of progesterone in promoting apoptosis of ovarian cancer cells as reported by Modugno et al. (2012) [21].

On the contrary, the current work did not find a significant correlation between the different histopathological subtypes and OS in contrast to the same previously mentioned studies that found that OS was better for immunoreactive type and worse in mesenchymal type cases [7, 8]. This may be due to the considerable number of cases that died because of the disease during the study period.

Longer OS in the current work is found to be significantly correlated with primary debulking surgery followed by adjuvant treatment as well as the use of combined taxol carboplatin-based chemotherapy. Similar findings are reported by Bristow and Chi (2006) and Murakami et al. (2016) [7, 28], but in contrast to the result of a recent study of Machida and colleagues [29].

Our work presented a detailed analysis for the histopathological features of HGSOC with evident histopathological findings that allowed categorization of cases into groups parallel to the molecular subtypes, with some of these features (the proliferative and immunoreactive subtype) are supported by IHC markers taking into consideration the management protocol that the patient received. These groups showed significant prognostic differences in terms of PFS that indicate the value of applying histopathological features during the evaluation of HGSOC and no longer considering it as a single tumor type.

On the other hand, the relatively small number of cases enrolled in the study limited us. This was due to the necessity for the selection of cases with the complete clinical and follow-up data. Additionally, the death of many cases during the study period hindered achieving a significant correlation with overall survival.

## Conclusion

There are histopathological differences in HGSOC that can help in its subtyping. These are supported by IHC only for proliferative and immunoreactive subtypes. These subtypes do have prognostic relevance.

## Data Availability

Available.

## Abbreviations

HGSOC: High grade serous ovarian carcinoma
TCGA: The cancer genome atlas
EMT: Epithelial mesenchymal transition
IHC: Immunohistochemistry
H&E: Hematoxylin & Eosin
TILs: Tumor infiltrating lymphocytes
SET: Solid endometroid transitional
NACT: Neoadjuvant chemotherapy
TMA: Tissue microarray
PFS: Progression-free survival
LVI: Lymphovascular invasion
OS: Overall survival
